# Ultrastructure and Light Microscope Analysis of Intact Skin after a Varying Number of Low Level Laser Irradiations in Mice

**DOI:** 10.1155/2014/506051

**Published:** 2014-01-29

**Authors:** Mamie Mizusaki Iyomasa, Juliane Caroline Leão, Élen Camargo Rizzi, João Paulo Mardegan Issa, Fernando José Dias, Ii-sei Watanabe, Daniela Mizusaki Iyomasa

**Affiliations:** ^1^Department of Morphology, Physiology and Basic Pathology, Faculty of Dentistry of Ribeirao Preto, University of Sao Paulo, Avenida Café, s/n, Monte Alegre, 14040-904 Ribeirão Preto, SP, Brazil; ^2^Department of Anatomy, Institute of Biomedical Science, University of Sao Paulo, São Paulo, Avenida Prof. Lineu Prestes, 2415, 05508-000 Butantã, SP, Brazil

## Abstract

Low level laser therapy (LLLT) has been used to relieve pain, inflammation, and wound healing processes. Thus, the skin is overexposed to laser and this effect is not completely understood. This study analyzed the effects of the number of laser applications (three, six, and 10) on the intact skin of the masseteric region in mice of strain HRS/J. The animals (*n* = 30) were equally divided into control (0 J/cm^2^) and irradiated (20 J/cm^2^), and each of these groups was further equally divided according to the number of laser applications (three, six, and 10) and underwent LLLT on alternate days. Samples were analyzed by light microscopy and transmission electron microscope (TEM). The animals receiving applications exhibited open channels more dilated between the keratinocytes and photobiomodulation effect on endothelial cells and fibroblasts by TEM. Under the light microscope after 10 laser applications, the type I collagen decreased (*P* < 0.05) compared to the three and six applications. Under these experimental conditions, all numbers of applications provided photobiomodulatory effect on the epidermis and dermis, without damage. More studies are needed to standardize the energy density and number of applications recommended for laser therapy to have a better cost-benefit ratio associated with treatment.

## 1. Introduction

The skin is the most voluminous organ, which covers the entire surface of the body [1]. As a complex organ, it has several functions such as preventing loss of water and electrolytes and acting as a barrier against injuries (mechanical, chemical, thermal, and sunlight damage) and thermoregulation [2]. The skin is constituted by three layers: epidermis, papillary dermis, and reticular dermis, composed respectively by squamous stratified epithelium, loose connective and connective containing compact collagen fibers [1]. It is more exposed to laser irradiation than other parts of the body since skin is the first structure to receive the incidence of this light [3].

According to the location, epidermis is thicker or thinner varying from 0.06 to 1 mm [2]. The most predominant cell type in the epidermis is the keratinocyte, a name derived from keratin, its main product. Several morphologically distinct epidermis layers are formed as the keratinocytes move from the basement membrane to the skin surface [1]. The dermis is constituted mainly by collagen and could be divided into two layers with no distinct limits: the upper papillary dermis and the lower reticular dermis. The papillary dermis is constituted by dermal papillae, which are conic projections interdigitated with epidermal rete ridges that increase the area of dermal-epidermal interface to provide mechanical anchorage and nutrition for the epidermis [1].

Dermatological researches have also reported experimental improvements associated with LLLT for facial rejuvenation treatment [4] and cutaneous wound healing [5]. However, as presented in the literature, different laser types, energy density, duration of treatment, and other parameters were used in the studies. Some researches show a faster skin repair, such as using the National Aeronautics and Space Administration (NASA) light-emitting diode (LED) 880 nm, delivering 4 J/cm^2^ and 50 mW/cm^2^ for 14 consecutive days [6]; the aluminum-gallium-indium-phosphorus (AlGaInP) diode laser 670 nm, 25 mW/cm^2^, irradiating the total daily energy density of 30 J/cm^2^ [7]; the helium-neon (He-Ne) laser, 632.8 nm, 10 mW, energy density of 1 J/cm^2^ irradiated on the 1st, 5th, 8th, 12th, and 15th day after-wounding [8]; the VR-KC-610 Laser, Dentoflex, CW, Brazil, l670 nm, 9 mW, 0.031 W/cm^2^, 4 J/cm^2^, single irradiation after surgery [5]; and the NAFL and AFL treatments which were performed with the same total energy of 12,000 mJ cm^2^, with four sessions and an interval of 3 weeks between sessions. So with all the variability used in these studies, it is difficult to compare different results.

While Whelan et al. [6] produced an increase in growth of 155–171% of normal human epithelial cells *in vitro* using the NASA LED (670–880 nm), other groups have failed to report similar successes. For example, using a 585 and 595 nm laser at energy densities of 10–30 J/cm^2^ delivered twice on each subject, Pikkula et al. [9] noted that the laser may be more harmful at 585 nm than 595 nm in the vascular response. In culture cell irradiation performed with the He-Ne laser at a wavelength of 632.8 nm, 2 mW/cm^2^, with a single irradiation exposure on two consecutive days, Hawkins and Abrahamse [10] noted that higher energy densities than 10 and 16 J/cm^2^ appeared to produce a significant amount of cellular and molecular damage. Basso et al. [11] on the other hand noted that cells irradiated with an InGaAsP diode laser (780 ± 3 nm; 40 mW) with energy densities of 7 J/cm^2^ every 24 h totaling three applications presented the lowest metabolic activity when compared with the nonirradiated control group. Thus, it seems that, in general, lower energy density is better than higher one for cell viability. Despite these indications, more studies are required to investigate parameters appropriate for each therapy.

Although several studies have investigated the energy density used in therapy of deep structures such as the temporomandibular disorders (TMD) and orofacial muscle pain [12–14], few studies have examined the impact of these energy densities on the skin. By this way, our study used animal models that have the characteristics of human skin to investigate the effect of a number of applications (three, six, and 10) of low level laser (LLL) on the intact skin of mice, strain HRS/J, using an energy density usually recommended for clinical procedures such as dysfunction of the stomatognathic system.

## 2. Materials and Methods

### 2.1. Animals and Treatment

The HRS/J strain mice were selected to perform this study because of their hairless particular characteristic which makes it unnecessary to shave the mice skin avoiding skin damage, also because of its similarity to human skin. Thirty male mice, weighing ~35 g, were obtained from the vivarium of the Faculty of Dentistry Ribeirão Preto, University of São Paulo (USP). The animals were maintained in polyethylene boxes under controlled room temperature conditions between 24°C and 25°C, with 12 h of light. They received food and water *ad libitum*. All procedures of this study were approved by the Local Ethics Committee (number 7.1.879.53.1) in accordance with international laws of animal use.

Mice were randomly divided into two main groups: irradiated group (I) (*n* = 15) and control group (C) (*n* = 15). Each group was subdivided according to the number of laser applications (three, six, and 10). The laser was applied on alternated days (Table 1). Preceding the irradiation, mice were anesthetized with halothane and irradiated by the MM Optics Twin Laser apparatus (frequency of 56/60 Hz, 100~240 V, maximum optical power of 70 mW, wavelength of 780 nm, maximum energy density of 315 J/cm^2^, gallium-aluminum-arsenide semiconductor) at an energy density equivalent to 20 J/cm^2^ (continuous wave, 40 mW, 20 s, spot area 0.04 cm^2^) on skin of the middle region of the left masseter muscle [15].

The control group also received the same type of treatment, but with no irradiation. Seventy-two hours after the last application [16], the animals were anesthetized with intramuscular injections of xylazine (10 mg/Kg) and ketamine overdose (150 mg/Kg) and then perfused with buffered formalin. The masseteric region skin was dissected and divided into small portions for transmission electron microscopy and light microscopy for evaluation of collagen by picrosirius staining.

### 2.2. Transmission Electron Microscopy

The skin was cut into fragments and immediately fixed with 2.5% glutaraldehyde in 0.1 M phosphate buffer for 2 h at 4°C, followed by postfixation with 1% osmium tetroxide. The fragments were contrasted with 0.4% uranyl acetate overnight, dehydrated with a graded alcohol series, and then embedded in Spurr resin (Electron Microscopy Sciences, Hatfield, PA 19440, USA). The blocks were sectioned with 1–3 *μ*m thickness using a Reichert ultramicrotome glass knife and stained with toluidine blue solution in 50% ethanol to determine the area of interest. Finally, ultrathin (90 nm thick) slices obtained with a diamond knife were collected using a 200-mesh grid and contrasted with 4% uranyl acetate solution and lead citrate 0.4%. All slices were examined under a JEOL 1010 transmission electron microscope. Data from transmission electron microscopy were qualitative description of changes in the morphology of the cells and the organelles correlated.

### 2.3. Light Microscope

The remaining portion of the skin of the middle region of the left masseter muscle was immersed in 10% formaldehyde and processed in paraffin. From each block, 6 *μ*m thick slices were made and stained with picrosirius, which is specific for collagen analysis [17]. Five fields were randomly selected among the section obtained from each block, and the images were captured in polarized light with a Leica DC 300F camera adapted for the DMLB2 Leica microscope, with the aid of the Leica IM50 program, installed on a connected computer. After the capture, these images were processed in ImageJ software, in which the color channels were splitted for quantification of collagen I, using the red channel of the image. This image obtained from the red channel was converted into binary (black and white) and the quantification was performed using the “area calculator” of ImageJ. In addition, the skin area of each image was circulated and by this way the total area of the skin was obtained. The value presented as a percentage of the study is given by the ratio of the red area (collagen I) in binary to the total area of the skin. After being captured, images were analyzed by an investigator blinded to the treatment.

### 2.4. Statistical Analysis

For quantitative analysis using light microscope photomicrographs (picrosirius staining), data were statistically analyzed by the ANOVA and Holm-Sidak posttest using SPSS software, version 17.0, for Windows (SPSS Inc., Chicago, IL, USA) (*P* = 0.05). These data were presented as mean ± SD and all groups were compared one to each other (intra- and extragroup comparisons).

## 3. Result

### 3.1. Transmission Electron Microscope

**Figure 1:**
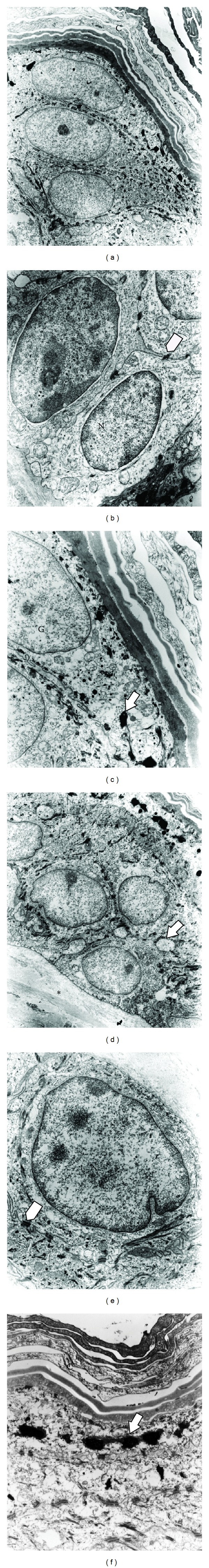
(a)–(c) Control group. (a) Epidermis with keratinocytes (*) and corneum (c) layer (×4000). (b) Basal cell layer with loose chromatin nuclei (n), joined by desmosomes (arrow head) showing intermediate keratin filaments (×10000). (c) Granular cell layer (g) with keratohyalin granules (arrow) surrounded by ribosomes and thick electron dense plasm a membrane (*) (×10000). (d)–(f) Irradiated group. (d) Epidermis (e), the dermis (*), and dilated channels (arrow) between keratinocytes of the metabolically active layer (×4000). (e) Basal keratinocytes layer jointed by desmosomes (arrow head) showing the nucleus (n) and bundle of keratin fibrils (×10000). (f) Granular keratinocytes layer with abundant keratohyalin granules (arrow), thick electron dense plasma membrane (*) with vesicles suggesting lamellar granules exuding their contents, and corneum layer keratinocytes (c) (×10000).

**Figure 2:**
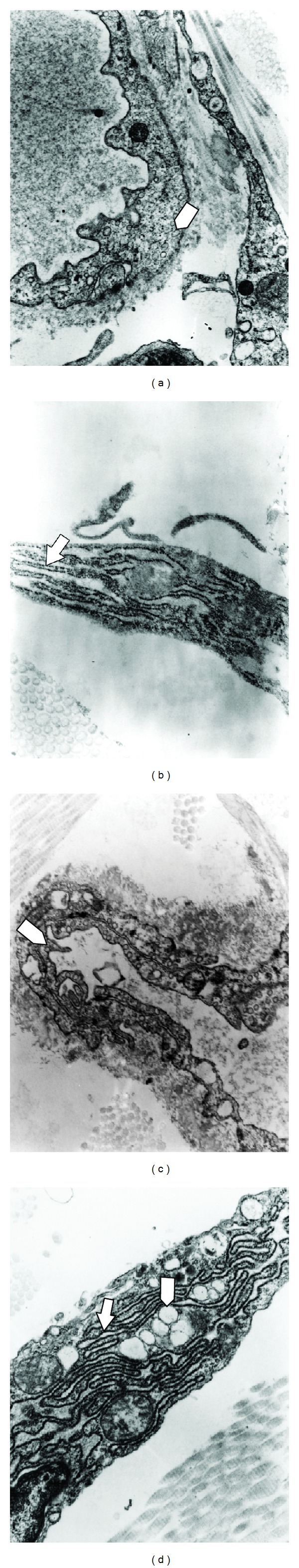
(a)-(b) Control group. (a) Endothelial cell (arrow head) with pinocytotic vesicles (×20000). (b) Fibroblast processes showing granular endoplasmic reticulum (arrow) (×20000). (c)-(d) Irradiated group. (c) Endothelial cells (arrow head) with pinocytotic vesicles and their cytoplasmic protrusion (×20000). (d) Fibroblast processes with abundant granular endoplasmic reticulum (arrow) and secretory granules (arrow head) (×20000).

The epidermis (Figure 1(a)), which forms the uppermost multilayered compartment of the skin, was formed mainly of keratinocytes. All the control groups (C3, C6, and C10) showed similar basic features between groups. The keratinocyte cells of the basal and spinous layer were joined by desmosomes or interdigitations (Figure 1(b)), showing large nuclei with loose chromatin, the presence of mitochondria, and scarce intermediate keratin filaments and tonofibrils, among other organelles (Figure 1(b)). In the granular layer, the cells changed their shape and size, the nucleus became elongated, and cytoplasm revealed granules of keratohyalin surrounded by ribosomes and some bundles of intermediate keratin filaments, with a thick electron dense plasma membrane at the superficial face of the cell (Figure 1(c)). The cells become extremely flattened in the stratum corneum with a predominance of intermediate keratin filament bundles interspersed with amorphous material (Figure 1(c)). The lamina propria showed cytoplasmic processes of fibroblasts with granular endoplasmic reticulum (Figure 2(b)) and endothelial cells with some pinocytotic vesicles (Figure 2(a)).

The group irradiated with three, six, and 10 (I3, I6, and I10) applications showed the epidermis (Figure 1(d)) with general aspects similar to the control group, but in the metabolically active layer of the epidermis, some open dilated channels were noted (Figure 1(d)) after six applications. The keratinocyte cells of the basal layer showed in the cytoplasm rounded nucleus, mitochondria, and keratin fibrils densely distributed and were joined to each other by desmosomes and interdigitations (Figure 1(e)). In the granular layer, the flattened cells showed more abundant keratohyalin granules, mainly after 10 laser applications, with a thick electron dense plasma membrane at the superficial face of the cell showing some vesicles, suggesting lamellar granules exuding their contents into the intercellular space (Figure 1(f)). The cells of the corneum layer were found to be much flattened and showed a predominance of intermediate keratin filament bundles (Figure 1(f)). The lamina propria showed the cytoplasmic processes of fibroblasts rich in granular endoplasmic reticulum with dilated cisterns containing secretory granules after six and 10 applications (Figure 2(d)). The endothelial cells of the capillary showed a large number of pinocytotic vesicles and cytoplasmic protrusions facing the blood vessel lumen (Figure 2(c)).

### 3.2. Light Microscope—Quantitative Analyses

#### 3.2.1. Analysis of Control and Irradiated Groups

**Figure 3:**
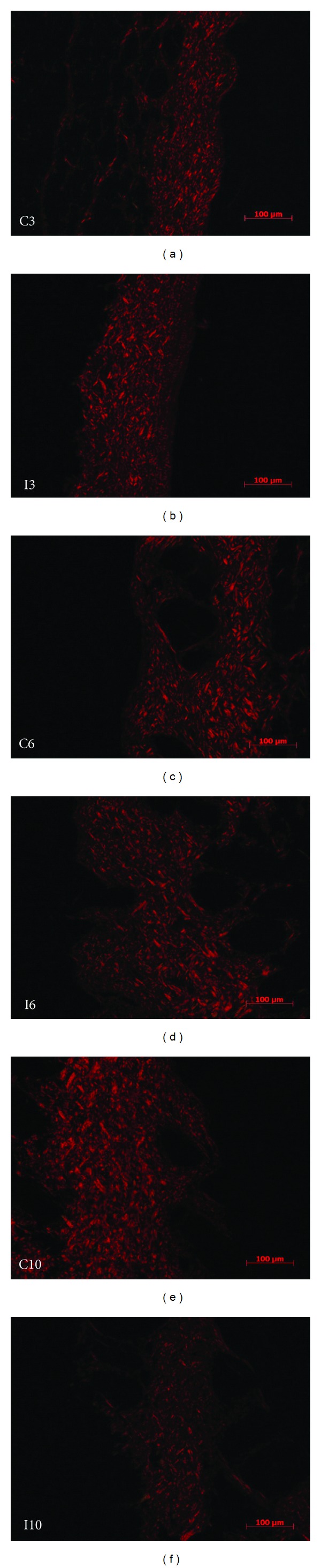
Collagen of the derm stained by picrosirius in the irradiated groups (I3, I6, and I10) and their respective controls (C3, C6, and C10).

**Figure 4:**
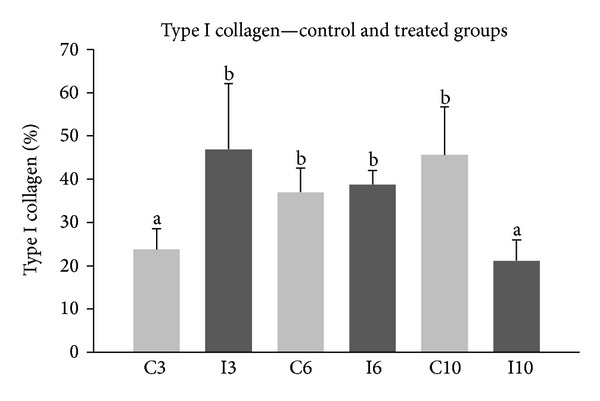
The comparison of type I collagen between irradiated groups (I3, I6, and I10) and their respective controls (C3, C6, and C10).

Figures 3 and 4 show the dermis images stained with picrosirius captured in polarized light of the control groups (C3, C6, and C10) and irradiated groups (I3, I6, and I10). In the group which received three applications of laser, there was a significant increase (*P* < 0.05) in the amount of collagen (46.87 ± 15.21%) compared to the control group (23.74 ± 4.75%).

In the group of six applications, the density of collagen was 36.94 ± 5.63% and 38.73 ± 3.26% in the control and irradiated groups, respectively, showing no significant differences (*P* > 0.05) between both groups.

With 10 applications, there was a significant decrease (*P* < 0.05) in the density of collagen (21.16 ± 4.76%) in the irradiated group compared to the control group (45.60 ± 11.07%).

#### 3.2.2. Analysis between Groups of 3, 6, and 10 Applications of LLL and Control Groups

In the analysis between irradiated groups, there was a significant decrease (*P* < 0.05) for groups of 10 laser applications (21.16 ± 4.76%) compared to the three and six groups, which were 46.87 ± 15.21% and 36.94 ± 5.63%, respectively. There was no difference between the groups of three and six. Interestingly, comparing the control groups receiving three, six, and 10 simulated applications, a steady increase in the quantification of collagen was noted; however, the difference between the groups with six and 10 simulated applications was not significant.

## 4. Discussion

The ultrastructural analysis of keratinocytes after three, six, and 10 laser applications revealed an earlier appearance of keratin fibrils (tonofibrils) in the basal layers and more abundant keratohyalin granules increase, according to the increase in the number of applications. Candi et al. [18] described that keratohyalin granules contain profilaggrin, the precursor of filaggrin, which aggregates the keratin filaments into tight bundles, forming an important scaffold for the subsequent maturation of the corneocytes. So, the early organization of keratin fibrils and abundant keratohyalin granules presented mainly in the epidermis irradiated with ten laser applications suggests an acceleration of the keratinization process. Studies by Leão et al. [19] corroborate this assertion, since after 10 laser applications, the authors observed a decrease in the basal layer, but no difference in the keratin layer area.

The thick electron dense plasma membrane at the superficial face of the granular cell layer showed some vesicles, suggesting lamellar granules exuding their contents into the intercellular space. This structure could represent the initial steps in the formation of the cornified envelope which, according to Hitomi [20], provides a barrier function at the outermost layer of the epidermis. The cornified envelope is a complex structure that involves the keratin filaments to aggregate into tight bundles at the same time of the synthesis of the other structural proteins crosslinked by transglutaminase enzymes, for terminal keratinocyte differentiation in the cornified layer [20]. Lamellar granules release glycolipid acil glicosilceramida in the intercellular spaces, in order to produce the waterproof plasma membrane described by Candi et al. [18] and Kierszenbaum [21]. We believe the vesicles to be the same lamellar granules which exude their contents into the intercellular space. Since the vesicles are present in both the irradiated and control groups, the experiment suggests that laser irradiation parameters do not alter the epidermis function of helping to avoid water loss.

Interestingly, the six irradiated groups with 20 J/cm^2^ showed more dilated open channels in the metabolically active layer of the epidermis. This fact could explain the increased area of the epidermis and its layers observed by optical microscope after six laser applications when Leão et al. [19] used the same conditions of our experiments. Due to dilation of open channels, it is possible that the nutrients diffusion has consequently increased the cells metabolism and activities, leading to a larger area of the epidermis with six applications of the used energy density.

All of the three irradiated groups (I3, I6, and I10) observed by the electron microscope had the fibroblast processes with abundant rough endoplasmic reticulum, which was dilated and had several secretory granules. This data confirms that the laser energy density in this experiment conditionally recommended for deep tissue therapy is able to photobiomodulate the fibroblast.

In the dermis of the irradiated and nonirradiated groups, the capillary endothelial cells showed pinocytotic vesicles indicating diffusion of nutrients and cellular activity. The cytoplasmic protrusions of the endothelial cell protruding into the blood vessel lumen suggested angiogenesis. Irradiation with LLL has also shown the proliferation of endothelial cells, contributing to an increase of angiogenesis and acceleration of wound healing *in vivo* in different tissue types [22–27]. Our data showed that the LLL using an energy density usually recommended for deep structure contributes to nutrients diffusion in all numbers of applications, without revealing damage.

When the collagen density was analyzed by light microscope, our results in the intact skin showed an increase with three and decrease with 10 laser applications, when compared with their respective control groups. Despite the increase in density of collagen fibers observed in the group of three laser applications, the difference was not significant compared to the control group of six and 10 and the irradiated group that received six applications, suggesting no formation of new collagen fibers in the intact skin. However, laser action in the treatment of tendons [28] and on skin wound healing therapy increased collagen fibers [5, 8, 17, 29, 30]. The difference between these results and those of our study may be explained by irradiation having been applied on intact skin of our animals, which have no need of a major renovation of collagen. However, the fibroblast was photobiomodulated by a number of applications and the intensity used in our protocol, according to the electron microscope analysis.

From the comparisons of the control groups (C3, C6, and C10), which received simulation of laser applications, the increasing density of collagen, according to the increase of the simulations, was observed and can be explained by the development of the skin, since the experimental animals were young. Le Guellec et al. [31] showed that in zebrafish the diameter of collagen fibers increases from 26 days of life. Furthermore, a study on the skin of mutant hairless USP mice showed an increase in thickness of the dermis during the study period (18 days and one, three, six, and eight months). However, the study did not analyze the collagen fibrils by the quantitative method [32]. This information corroborates the explanation of the gradual increase of collagen density observed in the C3, C6, and C10 groups. From the comparisons between the irradiated groups, there was a significant decrease in collagen density for the group of 10 laser applications. The ultraviolet B (UVB) irradiation suppresses transforming growth factor beta (TGF-*β*) signaling pathways that produce type I collagen [33]. Therefore, it is conceivable that 10 applications of laser energy at 20 J/cm^2^ may be too much for the mouse skin, which is thinner than human skin. However, the mechanism by which this laser energy density interferes with the decrease of collagen in the dermis of mice is still unknown and could be a different mechanism from that of UVB.

It is noteworthy that on the intact masseter muscle all of the different energy densities (0.5, 2.5, 5, and 20 J/cm^2^) used by Dias et al. [25] stimulated the expression of the matrix metalloproteinase and the oxidative metabolism, but they noted that 20 J/cm^2^ appeared too high for oxidative metabolism. Rizzi et al. [24] described better oxidative metabolism with six than 10 laser applications at 20 J/cm^2^ in intact masseter muscle in mice. Gonçalves et al. [34] observed an increase of the collagen with the GaAlAs laser (4 J/cm^2^ and 30 J/cm^2^), but no changes with the 60 J/cm^2^ on rat skin injury. So, these facts corroborate reports that the lower power laser has photobiomodulator potential [10, 34, 35]. On the other hand, higher energy densities cause morphological changes, reduced mitochondrial activity, and risks to DNA [10, 36] and can also induce apoptosis [37].

Laser therapy on clinical routine is widely used to relieve pain, inflammatory symptoms, and wound healing process. A better understanding of the impact of different LLLT numbers of applications/irradiations on the normal skin is crucial for a well-designed, nonharmful, and effective treatment.

## 5. Conclusion

Under these experimental conditions (20 J/cm^2^), three, six, and 10 laser applications provided photobiomodulation effect on intact epidermis and dermis of mice, strain HRS/J, without damage being revealed by electron microscopy. However, 10 laser applications decreased the type I collagen on the dermis. Thus, it is clear that more studies are needed to standardize the energy density and number of applications recommended for therapy of TMD to have a better cost-benefit ratio associated with treatment.

## Figures and Tables

**Table 1:** Mice's treatment frequency, energy density, energy deposited, and day of euthanasia per group.

Groups	Number of irradiations or simulations	Energy density (each irradiation) (J/cm²)	Energy (J)	Day of euthanasia
C3	3 simulations	0	0	Day 8
I3	3 irradiations	20	2.4	Day 8
C6	6 simulations	0	0	Day 13
I6	6 irradiations	20	4.8	Day 13
C10	10 simulations	0	0	Day 21
I10	10 irradiations	20	8	Day 21
